# Challenging perceptions about rural practice using narratives: a living library approach in medical education

**DOI:** 10.3389/fmed.2024.1452932

**Published:** 2024-12-10

**Authors:** Grace Perez, Rebecca Malhi, Kamiko Bressler, Melissa Monaghan, Aaron Johnston

**Affiliations:** ^1^Distributed Learning and Rural Initiatives, Cumming School of Medicine, University of Calgary, Calgary, AB, Canada; ^2^Department of Family Medicine, University of British Columbia, Vancouver, BC, Canada; ^3^Cumming School of Medicine, University of Calgary, Calgary, AB, Canada

**Keywords:** living library, library of life, human library, rural medicine, rural practice, attitudinal change, undergraduate medical education, pre-medical students

## Abstract

**Introduction:**

The shortage of physicians in rural Canada is a continuing challenge. Canadian medical schools have adapted strategies to increase the supply of rural physicians. This study appraises the effectiveness of the living library (also called Human Library©) in medical education, as an avenue for medical and pre-medical students to engage in dialogue with rural health professionals. Similar to a conventional library, readers check out books, except that “books” are human volunteers willing to share relevant personal experiences, and “readers” are the learners. The reading is the personal interaction between human books and students through narratives of experiences. The program “The Library of Life—Stories of Rural Medicine” (TLoL), was developed to provide students with better understanding of rural life and practice through narratives.

**Methods:**

This is a mixed methods study, using pre- and post-event surveys. Statistical comparisons were done using Wilcoxon and McNemar’s tests. Thematic analysis was used to explore students’ expectations of TLoL and to describe their experience and key takeaways.

**Results:**

Most of the participants were from an urban background, had low familiarity with rural medicine and only 44% would consider a rural career prior to TLoL. After TLoL, improvements were observed in: (i) envisioning rural medicine as career option (*p* = 0.009), (ii) appreciation of rural living (*p* = 0.013), (iii) need for rural physicians (*p* < 0.001). and (iv) rural practice consideration (*p* = 0.001). Themes from students’ motivations for participation were: (i) students’ curiosity, interest, and (ii) their willingness to engage in dialogue with the human books. Themes from the key takeaways were that TLoL allowed students: (i) to walk in a rural professional’s shoes, enabling them to see “rural” in a new light, and (ii) to self-reflect and gain a sense of personal growth.

**Conclusion:**

Students made gains in attitudes and perceptions toward rural practice. Narratives have the power to challenge held beliefs around rural practice and life, and can encourage students to consider things that traditional medical teaching may not. TLoL can be an effective learning modality in medical education to provide information about rural medicine, in combination with learning opportunities such as rural block rotations and longitudinal clinical clerkship immersions.

## 1 Introduction

This study explores the role of the Living Library approach in medical education. The Living Library concept (also known as Human Library©) was originally developed in Denmark in 2000 as a strategy to bring people together to challenge prejudice, with the program described as “a library of the people” (1). Its motto “to unjudge someone,” stemmed from a desire to overcome discrimination and remove stigma from social conditions such as homelessness, alternate lifestyles, disabilities, etc. (2). A Living Library offers a framework to bring people from different groups together and have respectful and meaningful dialogue. It mimics the format of a conventional library with readers borrowing books, except that the “books” are human volunteers willing to share relevant personal experiences, and “readers” are the participants (e.g., medical students) (3). The reading is the personal interaction between human books and readers through narratives of experiences and personal stories (4, 5).

With the persistent shortage of physicians, the provision of equitable healthcare services in rural and remote communities in Canada is an ongoing challenge, putting additional strain on the Canadian healthcare system (6). Using Statistics Canada’s definition of “rural” and “small town,” about 19% of Canada’s population is rural (7). Recent data from Canadian Institute for Health Information (CIHI) indicated 96,020 physicians present in Canada, representing 247 physicians per 100,000 population, but only 8% of family physicians and 2% of specialists are in rural practice (8). Less than 10 years previously, 14% of family physicians were in rural Canada (9). This downtrend has reduced rural access to family physicians and widened the disparity in rural–urban health outcomes (10).

Canadian medical schools have employed strategies to increase the likelihood of physicians entering rural practice (11). These include admissions policies, rural-oriented medical curriculum, rural practice learning experiences, faculty values and attitudes, advanced procedural skills training and rural student recruitment (12). Rural student recruitment is based on extensive research demonstrating that those who have social attachments to their rural origins are most likely to practice in rural settings (13–17). A study of graduates from a Canadian medical school determined that having rural educational experiences on the continuum from high school through medical residency were associated with rural practice intention (18) and further substantiated by the recent synthesis of factors associated with successful recruitment and retaining of doctors in rural areas (19, 20). While rural upbringing is the most important predictor for rural practice consideration (21), the numbers of medical students from rural areas are not sufficient to eliminate the shortage of rural physicians. Studies have revealed that sociodemographic characteristics of Canadian medical students are not representative of the general population (22, 23) with only 6.4% of learners having grown up in a rural area compared with 18.7% of the Canadian population (24) and it is further compounded by a steady decline in enrolment of medical students with rural origins (25). Thus, a substantial portion of physician supply for rural Canada will come from urban areas (26, 27). Research showed that incoming medical students from urban and rural areas differ in their perceptions of rural life and practice and expression of practice intention (28). Likewise, an evaluation of factors affecting future practice decisions of urban-reared medical learners revealed that most know little of rural life and their perceptions of rural medicine often relied on generalizations and stereotypes (27, 29). This is concerning because misconceptions and negative beliefs about rural medicine may dissuade urban-origin students from considering rotations in non-metropolitan locations or from eventual rural practice (3). In addition, feeling prepared for small-town living has been found to be a stronger indicator of physician retention than feeling prepared for rural practice (30). Hence, it is important that urban-origin learners are provided opportunities to gain an appreciation of rural life and learn more about rural practice, to encourage a robust pipeline of students who will consider future rural practice.

The authors of this paper are from a department of a medical school in Western Canada, aiming to provide rural educational opportunities and transformational experiences to encourage rural healthcare interest among medical students. Rural learning experiences include rotation-based clerkship in rural settings, longitudinal clinical rural immersions and the use of rural physicians as teachers and mentors. To better prepare the learners, the authors needed to find ways of making students consider things that traditional medical teaching may not, especially regarding perspectives about rural life and rural medical practice. The Living Library model was repurposed in a medical education context through their department program “The Library of Life—Stories of Rural Medicine” (TLoL), to create an avenue for students to interact with rural healthcare professionals and other professionals with rural interests (3). This concept has been increasingly adapted and proven valuable in university settings and health disciplines, including diversity training and intercultural education (31), reducing prejudice in healthcare delivery (32), promoting mental health literacy and recovery (33, 34), reframing attitudes in higher education (2), and developing cultural awareness and sensitivity among occupational therapy students (35). The authors hypothesized that listening to narratives from rural physicians and other health professionals would provide learners with personal connection and valid information that may encourage participation in their department’s rural training opportunities, help change perceptions about rural life and foster more favorable attitudes toward rural medical practice.

## 2 Methods

### 2.1 Event format

The authors organized three TLoL events. At each event, rural physicians and other professionals involved in rural medicine were recruited to discuss their career and personal experiences in rural communities with medical learners. Due to Covid-19 restrictions, the first event in 2020 was held online. By 2022, most of public health restrictions were lifted and two in-person TLoL offerings were held in 2022 and 2023. Each in-person TLoL followed a 3-rotations format, each rotation lasting 30 min: 15 min for books’ narratives, 10 min for interactive questions-and-answers portion and 5 min for transition. The Conjoint Health Research Ethics Board (CHREB) of the University of Calgary approved this research project.

### 2.2 Participants

#### 2.2.1 Human books

Family physicians and other professionals in rural practice and those with rural interests were recruited to be the human books. Persons with relevant and engaging stories and experiences were selected and asked to present narratives of their experiences on some aspects of their work and rural life. TLoL has 25 human books in catalog. Books varied by community location, type of practice and sociodemographic background including age, gender, length of practice, ethnicity, and completion of medical training from both international and Canadian medical schools. At each TLoL session, 9 human books were available to present their stories. Some books have participated at multiple TLoL events.

#### 2.2.2 Readers

The “readers” are medical students and pre-medical students from disciplines such as Nursing, Health Sciences, and other allied health programs who were invited to register. The authors advertised the event using posters and reached out to departments with similar rural interests for support spreading the word. Students who wished to participate as “readers” were asked to register by completing an online registration form. After registration, the “readers” were randomly allocated as equally as possible to the books.

### 2.3 Measures—mixed methods

During the registration process, a Qualtrics survey (Qualtrics 2020) was used to collect data on students’ level of familiarity and interest in rural practice. The survey collected demographic information (gender, nationality, age, rural origin, program of study and year in the program) and the students’ reasons and expectations for participating. After TLoL, the students completed another survey about their TLoL experience. To evaluate the impact on attitudes and perceptions to rural life and practice data were collected that will enable before-and-after comparisons. Pre- and post-TLoL data were linked by creating unique identifiers for each student, based on 3 items: (i) last 2 letters of their mother’s first name, (ii) year graduated from high school, and (iii) first 3 letters of their birth month.

The questions were presented slightly differently between the pre- and post-event surveys as a strategy to mitigate potential “response-shift bias,” which is a limitation of pre- and post-test designs (36, 37). Another strategy is methodological triangulation which was employed in this mixed methods study, by collecting both quantitative data and qualitative insights, so that the limitations from each method may be transcended by comparing findings from different perspectives (38, 39).

#### 2.3.1 Quantitative data about attitudes and perceptions

The students were asked to use 5-point Likert-type scales to rate their familiarity with rural medicine (*1 = not familiar at all to 5 = extremely familia*r), and their agreement to statements about aspects of rural medicine (*1 = strongly disagree, 2 = disagree, 3 = not sure, 4 = agree, 5 = strongly agree*): as potential career option, scope of practice, rural life and work, interest in rural healthcare, and need for rural doctors. The related statements were phrased slightly differently in the pre- and post-TLoL surveys. Statements before TLoL were: (i) *rural medicine would be a good fit for someone like me*, (ii) *the wide scope of practice in rural medicine makes for an interesting career path*, (iii) *there are benefits to practicing in a rural or remote community* (iv) *I’m interested in learning more about opportunities for rural medical practice*, and (v) *it would matter to consider a consider practicing medicine in a rural community*. Post-TLoL the statements were: (i) *medical practice in a rural community would be a suitable career option for me*, (ii) *having a broad scope of practice would be an interesting career*, (iii) *performing a* var*iety of skill sets would be appealing and rewarding*, (iv) *I would like more opportunities to connect with people in rural practice*, and (v) *I feel that rural physicians play a vital role in the community.* For future practice consideration, the students were asked whether “*they could see themselves engaged in a similar rural practice*” measured on a 3-point scale (*no, not sure, yes*).

To evaluate TLoL as a learning modality students were asked to rate their experience based on the following attributes using the same 5-point agreement scale as above:

- *It was a great venue to have personal contact with people in rural practice*
- *I enjoyed hearing about the life and career experiences of the human books*
- *It is a good way to learn more about the nature of rural medical practice*
- *It offered more insight about the lifestyle benefits in a rural community*
- *It made me learn more about myself and my interests*
- *It gave me an appreciation of the wide scope of practice in rural medicine*
- *It gave me a better sense of how rewarding rural medicine can be*
- *Overall, the library of life was a positive experience for me.*

All statistical analyses of the quantitative data were conducted using SPSS statistical software (IBM SPSS version 29). A nonparametric test, Wilcoxon test for paired observations, was employed to compare the related statements before and after TLoL. For rural practice consideration, which was measured categorically, McNemar’s test for change was used. To describe the acceptability of TLoL as a learning modality, the responses were summarized using counts and percentages. The statistical significance was set at the 5% level of error probability.

#### 2.3.2 Qualitative data about motivations, expectations, and key takeaways

Qualitative data were collected to characterize the motivations and expectations of students for attending TLoL through open-ended questions. Before TLoL, the questions were: “*what made you decide to participate in the event*” and “*what are you hoping to get out of attending*.” The responses were blended for both questions and the authors decided to combine the data for each participant to represent factors that influenced students’ participation (motivations and expectations). After the TLoL, data collected to understand students’ thoughts, attitudes, and perceptions used the questions: “*what were the take-home messages*” and “*reasons why or not they could see themselves engaged in a similar rural practice*.” The post-TLoL responses also had related patterns and concepts, responses to represent key takeaways and outcomes of TLoL were merged at the individual level, similarly to the pre-session data.

To derive a rich description of students’ thoughts and TLoL experiences, and what they gained from participation, thematic analysis was performed with conceptual coding (40–43). The analysis followed similar steps used by qualitative researchers working in the area of living library (33, 35): (1) familiarization with the data, where authors (GP, KB, MM) involved in the qualitative analysis read and re-read the responses to develop familiarity with what students are saying; (2) after familiarizing with the text, the authors separately identified meaningful words or phrases that form concepts or patterns (codes), and (3) authors reviewed the codes together to define common themes, (4) authors discussed to reach consensus, with a third author adjudicating as needed, and established a coding scheme, (5) results were then recoded using this coding scheme, and (6) review of themes and selection of quotes to reflect the themes.

Authors KB and MM coded the themes for pre-event data on motivations and expectations and determined relevant concepts and tabulated their frequencies. Post-event, GP and KB coded the responses for takeaways and outcomes from TLoL, while GP and MM worked on the overall TLoL experience. The authors aimed to reduce potential bias by having three people go through of steps 1 to 5 in determining the themes.

## 3 Results

### 3.1 Demographic profile of student “readers”

Data were only collected for the two in-person TLoL events. Student demographic characteristics are summarized in Table 1. There were 43 and 49 students in 2022 and 2023, respectively. Of the 92 total students, 74 (80%) gave their consent and provided usable data. Of 49 post-TLoL responses, 44 (90%) could be matched with a pre-survey response. The authors attributed the discrepancy to the fact that some registered students were no-shows, and that there were non-registered walk-ins who did not complete the pre- survey.

**Table 1:** Demographic profile of students.

Demographic characteristics	2022		2023		All	
	n	%	n	%	n	%
Students registered to attend	43		49		92	
Students providing data pre-event	35	100%	39	100%	74	100%
Students providing data post-event	20	57.1%	29	74.4%	49	66.2%
Gender						
Male	6	17.1%	6	15.4%	12	16.2%
Female	28	80.0%	32	82.1%	60	81.1%
No data	1	2.9%	1	2.6%	2	2.7%
Age group						
18–25 years	33	94.3%	28	71.8%	61	82.4%
26–30 years	1	2.9%	8	20.5%	9	12.2%
31–35 years	0	0.0%	2	5.1%	2	2.7%
No data	1	2.9%	1	2.6%	2	2.7%
Ethnicity						
Caucasian/European/White	8	22.9%	11	28.2%	19	25.7%
Black/African	3	8.6%	5	12.8%	8	10.8%
Latin American/Hispanic	1	2.9%	0	0.0%	1	1.4%
Middle Eastern/Arab/West Asian	5	14.3%	6	15.4%	11	14.9%
South Asian/East Indian	6	17.1%	7	17.9%	13	17.6%
Asian	8	22.9%	5	12.8%	13	17.6%
Filipino/Pacific Islander	1	2.9%	1	2.6%	2	2.7%
Metis	1	2.9%	1	2.6%	2	2.7%
Mixed ethnicity	1	2.9%	1	2.6%	2	2.7%
No data	1	2.9%	1	2.6%	2	2.7%
High school location						
Alberta—rural	6	17.1%	4	10.3%	10	13.5%
Alberta—regional	1	2.9%	1	2.6%	2	2.7%
Alberta—urban	26	74.3%	23	59.0%	49	66.2%
Other province—rural	0	0.0%	4	10.3%	4	5.4%
Other province—regional	1	2.9%	2	5.1%	3	4.1%
Other province—urban	0	0.0%	3	7.7%	3	4.1%
Other country—rural	0	0.0%	1	2.6%	1	1.4%
Rural or non-rural origin						
Rural	5	14.3%	10	25.6%	15	20.3%
Non-rural	28	80.0%	25	64.1%	53	71.6%
Not sure	2	5.7%	4	10.3%	6	8.1%
Program of study						
Doctor of medicine	8	22.9%	15	38.5%	23	31.1%
BS health sciences	13	37.1%	8	20.5%	21	28.4%
BS biological sciences	2	5.7%	3	7.7%	5	6.8%
BS neurosciences	2	5.7%	4	10.3%	6	8.1%
BS nursing	1	2.9%	0	0.0%	1	1.4%
Other sciences	9	25.7%	9	23.1%	18	24.3%
Program year						
Year 1	12	34.3%	21	53.8%	33	44.6%
Year 2	8	22.9%	5	12.8%	13	17.6%
Year 3	7	20.0%	7	17.9%	14	18.9%
Year 4	7	20.0%	5	12.8%	12	16.2%
Year 5	1	2.9%	1	2.6%	2	2.7%

Most (82%) students were 18–25 years old, 81% were female, with 66% graduated from high school in a metropolitan area and only 20% completed high school in a rural area. Regardless of high school location, only 20% consider themselves raised rurally and 6 students were not sure. The students were ethnically diverse and also represented Indigenous and immigrant populations. Almost one-third (31%) of students were in Doctor of Medicine program, while the rest were pre-medical students from other programs: Health Sciences (28%), Biological Sciences (7%), Neurosciences (8%), Nursing (1%), and other Sciences (24%). More than half (62%) were in the first 2 years of study in their program.

### 3.2 Quantitative data analysis

#### 3.2.1 Familiarity and interest to learn about rural medicine

**Figure 1:**
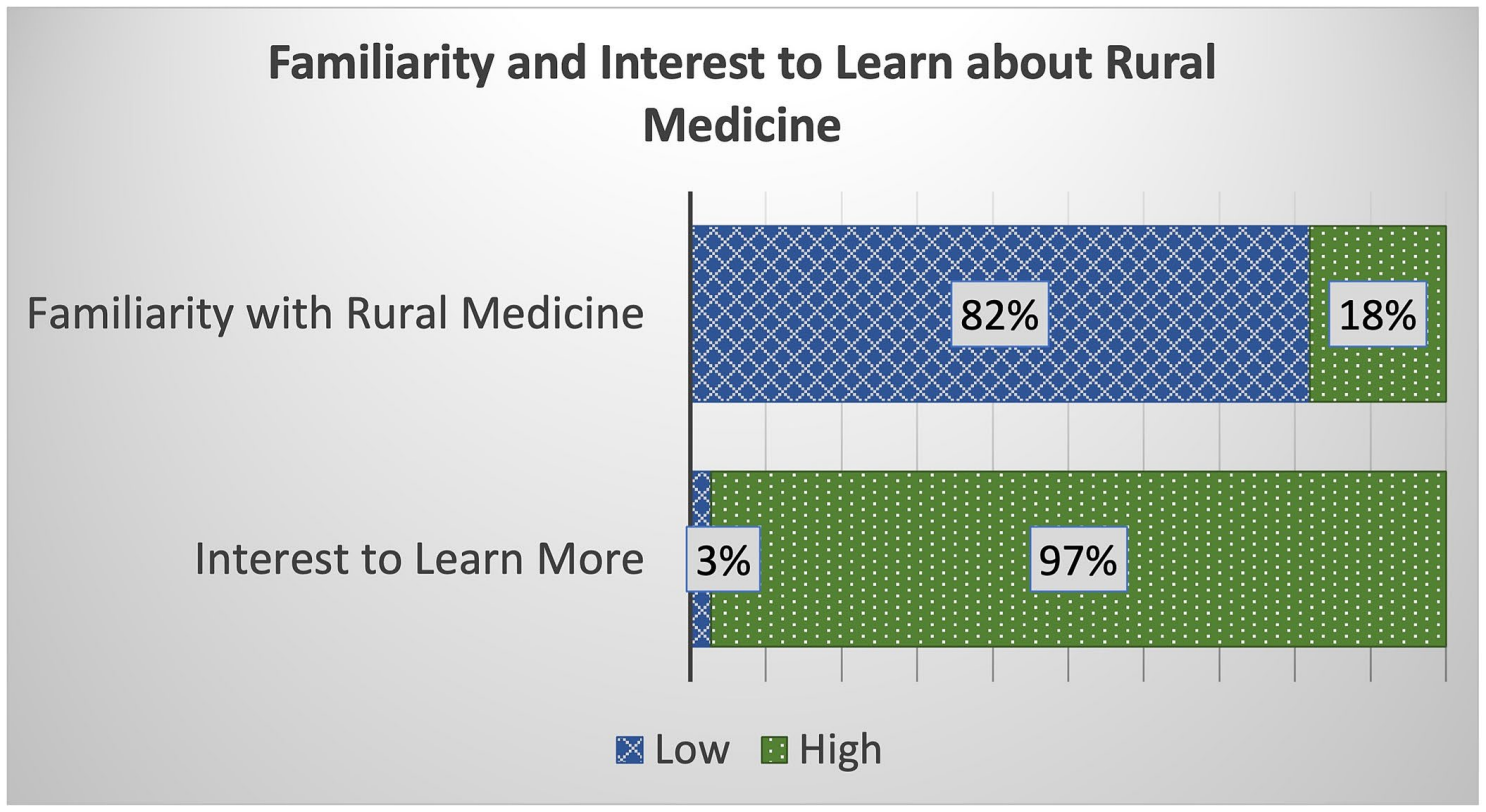
Distribution of student responses about their familiarity with and interest to learn more about rural medicine. The graph shows that most students have relatively low familiarity with rural medicine but almost all indicated strong interest to learn more about the topic.

Majority (82%) of students have relatively low familiarity about rural medicine; of the 72 students, 41 (57%) reported being “just a little” or “not familiar at all” with rural medicine, and 18 (25%) said “somewhat familiar.” Only 13 students (18%) were “familiar” to “extremely familiar” (Figure 1). The mean score was 2.51 (SD = 1.035). On the other hand, 70 (97%) of students indicated interest to learn more about rural medical practice.

#### 3.2.2 Attitudes and perceptions about rural medicine

Of the 49 students who completed the post-TLoL evaluation, 48 responded to the perceptions questions. The responses to attitudes and perceptions regarding rural medicine as future career option, scope of practice, rural life and work, interest in rural healthcare, and need for rural doctors, are summarized in Table 2. The authors grouped together “*strongly disagree*” and “*disagree*” responses and likewise combined the “*strongly agree*” and “*agree*” responses. The authors also considered the midpoint score “*not sure*” responses as non-favorable ratings.

**Table 2:** Attitudes and perceptions of students about rural medicine.

Attitudes and Perceptions about rural medicine	Strongly disagree/Disagree		Not sure		Strongly agree/Agree		Total
	n	%	n	%	n	%	
Before TLoL							
Rural medicine would be a good fit for someone like me	8	12%	32	44%	32	44%	72
The wide scope of practice makes for an interesting career path	4	6%	6	8%	62	86%	72
There are benefits to practicing in a rural or remote community	2	3%	7	10%	63	88%	72
Interested to know more about opportunities for rural medical practice	2	3%	0	0%	70	97%	72
It would matter to consider practicing medicine in a rural community	4	6%	18	25%	50	69%	72
After TLoL							
Rural medical practice would be a suitable career option for me	1	2%	19	40%	28	58%	48
Having a wide scope of practice would be an interesting career	2	4%	3	6%	43	90%	48
Performing a variety of skill sets would be appealing and rewarding	2	4%	2	4%	44	92%	48
Would like more opportunities to connect with people in rural practice	1	2%	2	4%	45	94%	48
I feel that rural physicians play a vital role in the community	1	2%	1	2%	46	96%	48

Before TLoL, students gave the lowest ratings to “*rural medicine would be a good fit for me*” [*n* = 32 (44%), mean = 3.49] and “*it would matter to consider practicing medicine in a rural community*” [*n* = 50 (69%), mean = 3.96]. After TLoL, more favorable ratings were provided for “*rural medical practice would be a suitable career option for me*” [*n* = 28 (58%), mean = 3.88] and “*I feel rural physicians play a vital role in the community*” [*n* = 46 (96%), mean = 4.79].

Attitude and perception scores of students improved after TLoL, across all the statements evaluated. The improvements in scores were confirmed by Wilcoxon signed rank tests for paired observations, for three aspects: (i) envisioning rural medicine as future career option (*p* = 0.009), (ii) appreciation of rural life and work (*p* = 0.013), and (iii) recognizing the need for rural physicians (*p* < 0.001) (Table 3).

**Table 3:** Pre- and post-TLoL comparisons of attitudes and perceptions scores.

Attitudes and Perceptions about rural medicine		N	Mean	SD	Median	Test statistic	*P*-value^a^
Future career option: Rural medicine would be good fit for me	Pre	47	3.49	0.856	3	−2.604	0.009*
	Post	48	3.88	0.959	4		
Scope of practice: Having wide scope of practice would make for an interesting career	Pre	47	4.15	0.884	4	−1.079	0.072
	Post	48	4.40	0.869	5		
Rural life and work: There are benefits to living and practicing in a rural community	Pre	47	4.11	0.729	4	−2.497	0.013*
	Post	48	4.42	0.846	5		
Rural healthcare interest: Interested in opportunities to connect with people in rural practice	Pre	47	4.45	0.717	5	−1.069	0.285
	Post	48	4.50	0.772	5		
Need for rural doctors: Rural physicians have vital and important role within the rural community	Pre	47	3.98	0.872	4	−4.238	<0.001*
	Post	48	4.79	0.683	5		

a *p-values based on Wilcoxon Signed Rank Test for paired observations; 44 paired observations tested.*

*Statistically significant.

#### 3.2.3 Attitudes toward rural practice

Post-TLoL, students were asked whether “*they could see themselves engaged in a similar rural medical practice*,” measured as “*no/not sure/yes*,” but the responses received were only either “*not sure*” or “*yes*.” To assess a change in attitudes to rural practice, this was compared to a surrogate variable, “*rural medicine would be a good fit for someone like me*,” as this naturally connected to practice consideration. To harmonize the measurements, the “*agree/strongly agree*” responses were recoded as “*yes*,” and all other responses as “*not sure*” (Table 4). An improvement was found in the proportion of students that would consider rural practice after TLoL, i.e., 33/46 (72%) versus 32/72 (44%), and was confirmed statistically significant using McNemar’s test for change (*p* = 0.001).

**Table 4:** Pre- and post-TLoL comparisons of attitudes to rural practice.

Attitudes toward rural practice	Not sure		Yes		Total
	n	%	n	%	
Pre: Rural medicine would be a good fit for someone like me	40	56%	32	44%	72
Post: I can see myself in a similar rural medical practice	13	28%	33	72%	46
McNemar’s test for change test result (44 paired observations)	*p*-value = 0.001*				

*Statistically significant.

#### 3.2.4 The library of life as learning modality

**Figure 2:**
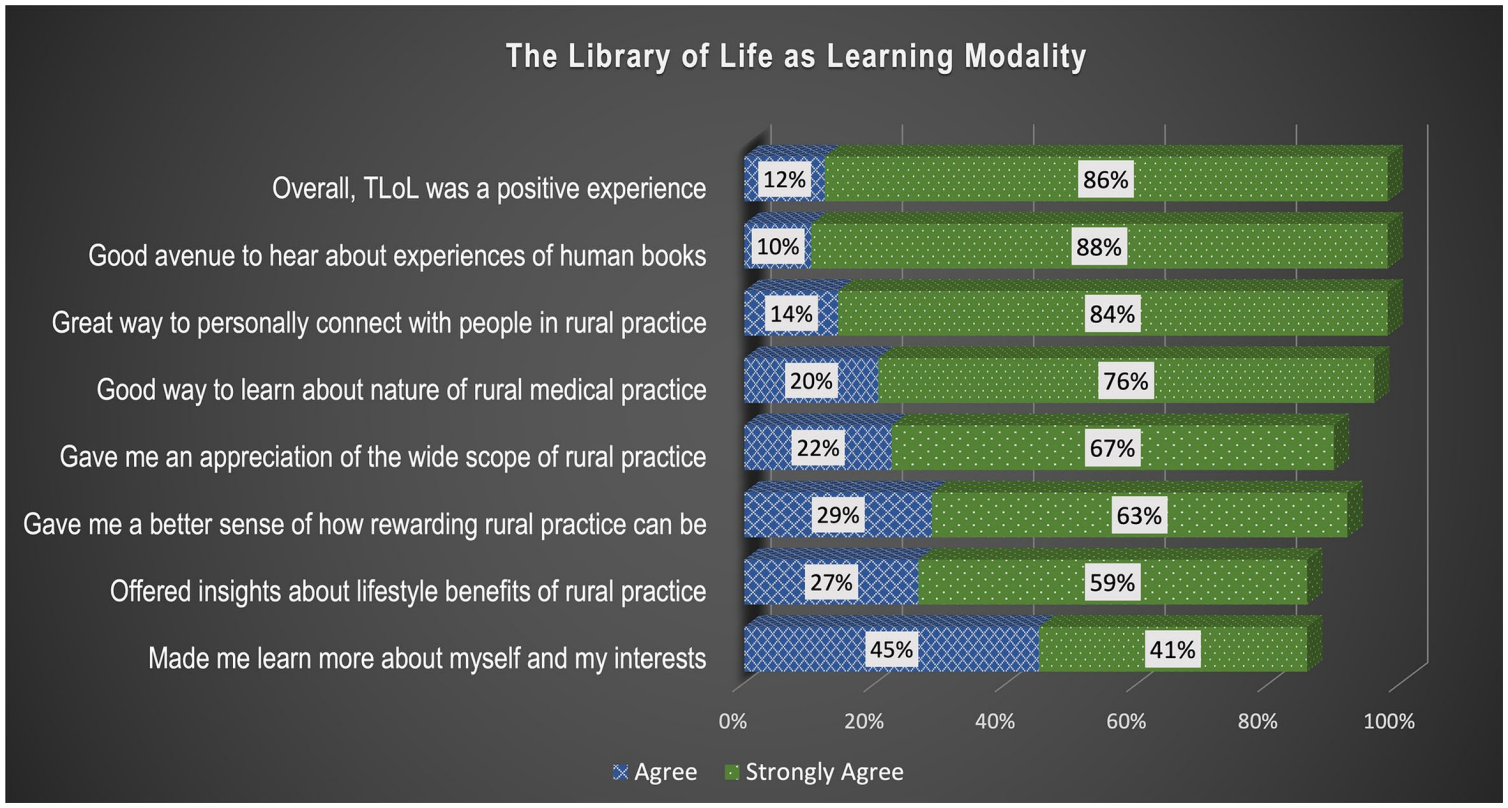
Distribution of student responses about their experience with TLoL. The graph depicts that almost all students provided high ratings to the attributes of TLoL, substantiating the convincing acceptability of TLoL as a learning modality.

Most students (48/49 or 98%) said that their TLoL experience was positive overall and rated all attributes fairly high (Figure 2). Highest rated attributes (95% up) indicated that TLoL was: (i) *a good way to have personal contact with people in rural practice*, (ii) *a good way to learn more about the nature of rural practice* and (iii) *where students enjoyed hearing about the life and career experiences of books*. The last attribute “*it made me learn more about myself and my interests*” still scored relatively high (42/49 or 86%).

Students’ additional comments provided more insight into their experience with TLoL, such as:

- *“I grew up in a big city and thus have not really considered practicing in a rural area. Most of the rural physicians I met were people who grew up in small towns, therefore I understand why they would want to also practice in a small town. However, I wish there could have been more “books” who grew up in large cities that chose to practice in rural areas. I feel that would be an interesting perspective to listen to!”* (S28)
- *“I wish something in this regard was integrated into the medical school curriculum.”* (S35)
- *“Being able to not only listen to but engage with the “books” and ask them questions was great to gain a better understanding of what rural medicine is like.”* (S16)
- *“I would have never considered a career in rural medicine whereas now it is definitely something I’m considering. All of the books were incredible to hear from and we are all so open and encouraging. I think that overall this was a very rewarding event and would be worth it for anyone to attend, regardless of whether or not they have an initial interest in rural medicine.”* (S60)
- *“Loved hearing perspectives from actual doctors. It is so much more impactful than reading a statement online and I loved that I got to ask questions.”* (S2)

### 3.3 Qualitative data—thematic analysis

#### 3.3.1 What made you decide to participate in TLoL and what are you hoping to get out of it?

From 73 responses collected, thematic analysis found two overarching themes: (i) students’ decisions to participate and their expectations of TLoL were aligned with the concept and purpose of the original Human Library©, and (ii) students demonstrated an open-minded curiosity, interest, and willingness to engage with and learn from the human books.

##### 3.3.1.1 Theme 1: students’ decisions to participate and their expectations of TLoL were aligned with the concept and purpose of the original human library©

- Students were excited to hear about books’ personal experiences, challenges and struggles.
  - *“I want to know more from folks who have tangible experiences. To get a better idea of lived experiences.”* (S67)
  - *“I am interested to hear about physicians’ experiences in rural medicine since I am considering this career path. I want to know whether people feel that practicing rural medicine made them better doctors. I am also interested in understanding some challenges about rural medicine.”* (S42)
  - “*I am hoping to gain a deeper understanding of the tribulations, joys, and realities of practicing rurally in Alberta.”* (S68)
  - “*I have always been interested in hearing people’s stories, and I believe it’s one of the most enriching ways to learn.”* (S71)
  - *“I’m really interested in individual stories and use other people’s stories and inspiration for how I should pursue my own goals.”* (S73)
- Students wanted to meet people and make connections.
  - *“The opportunity to engage with professionals in my field of interest and those studying in years ahead of me … I hope to gain some knowledge and advice regarding the years ahead of me while simultaneously making connections and meeting new people with similar passions, goals, and career paths.”* (S38)
  - *“I want to meet more cool rural physicians… and hopefully getting new mentors.”* (S9)
  - “*I am also hoping to build or enter a network of rural physicians for future opportunities to shadow.”* (S68)
  - “*Make connections to further explore this area of medicine.”* (S77)
- Students appreciated and welcomed the open and relaxed space of TLoL.
  - *“I am very interested in rural medicine and would love to learn more in a relaxed and open atmosphere.”* (S76)
  - “*I like the approach to the event that you can learn as much from people as you can a book. I also like that it is geared towards rural experiences.”* (S68)
  - *“I did not feel like I was being lectured at but rather having an open conversation so I felt like I was more engaged and learned more.”* (S11)
- Students looked forward to engaging with books and have an open dialogue.
  - *“Since I am a first year, RIME [Re-Imagining Medical Education] has done a good job of incorporating more story-telling from physicians and patients. The small taste of it I have had has left me wanting to learn more. I am going in with the hopes of learning more about life as a physician, especially in the rural context. I do not have any family or friends in the profession, so my knowledge was already quite limited… In essence, I hope to gain some more insight and wisdom about the field of medicine to inform my knowledge but more importantly, my practice.”* (S71)
  - *“I wanted to go deeper into tactical lessons and skills. Beyond the surface level especially as someone with rural experience already. I would have liked to gain tangible skills from this session. Or heard stronger opinions from the human books. I can appreciate that they want to be sensitive and demonstrate awareness of the unknowns, but it would have been more beneficial to have gotten insight into some of their stronger beliefs or experiences.”* (S96)
  - *“Diverse perspectives on rural medicine- the good and the bad. I would also love to hear from people who grew up in an urban setting and now practice rurally.”* (S76)

##### 3.3.1.2 Theme 2: students demonstrated an open-minded curiosity, interest, and willingness to engage with and learn from the books

- Students wanted to learn more about rural healthcare and valued the chance to discuss with rural practitioners.
  - *“The chance to discuss challenges rural physicians face and learn more about Healthcare. A deeper understanding and appreciation for physicians, the Healthcare system, and current gaps.”* (S11)
  - *“I wanted to broaden my knowledge of the role of healthcare in different communities, and how it can be made more effective to serve the community as a whole. I’m very interested in sociology of healthcare. More insight on healthcare in different communities, and potential solutions to make healthcare more accessible for everyone.”* (S21)
  - *“I’m really interested in rural medicine and generalism and really do see myself practicing in that space. I’d love to talk more with rural generalists about their practices!”* (S70)
- Students looked forward to hearing in different urban–rural perspectives, as most (80%) of our participants were from non-rural origins.
  - *“Would be interesting to hear about different perspectives about working in a rural environment, especially as someone coming from a non-rural background.”* (S4)
  - *“Living in Calgary my whole life I’ve had no exposure to rural living or rural medicine. I have heard such wonderful things about rural medicine so far and want to continue to learn more about what rural medicine is all about. Further information about what it is like to work and live rurally (especially from some of the rural docs who were not original from small towns).”* (S77)
  - *“While I have experience living in rural BC [British Columbia], I do not in Alberta and I am trying to gain a better idea of what that might be like.”* (S68)
- Students participated for personal advancement reasons, as encouraged by friends and other groups with similar rural interest
  - *“I decided to participate in the Human Library event as part of the enrichment aspect of the Pathways to Medicine program. I am hoping to make connections and learn more about the unique experiences of rural medicine.”* (S16)
  - *“A better understanding of rural medicine and the multifaceted role of a rural physician.”* (S4)
  - *“As I am planning a career in medicine, I was directed to this event with the help of the pathways to medicine scholarship to gain insight into rural medicine and explore more options than what I am currently aiming for.*” (S24)
  - *“Highlights of the main characteristics of the presenters careers and see if they are things I value/want to have in my life. Also understanding that if I do not practice rurally, how to support rural colleagues.”* (S69)
- Students looked forward to seek professional advice and guidance from the books, especially about what it takes to be a rural professional, as they consider their career options.
  - *“Unsure of what I want to do or where I want to work so trying to get more information and advice.”* (S69)
  - *“I wanted to get an insight into the lives and journeys of professionals as well as receive some advice on what I can do through my own journey.”* (S34)
  - *“I hope to gain more knowledge and guidance about pursuing rural medicine, as well as network with incredible individuals.”* (S72)
  - *“Learn about the experience of being a doctor working in rural and less serviced areas in Alberta. I hope to gain better insight as to what it means to be a rural physician.”* (S24)
  - *“Understanding a practicing physician’s continued motivations in medicine, how they navigate uncertainty, professional and personal boundaries, what qualities and actions they consider to be a good doctor.”* (S73)

#### 3.3.2 What were the take-home messages, and could you see yourself in a similar rural practice?

From 47 responses, thematic analysis revealed two overarching themes: (i) TLoL enabled students to see the “rural” context in a new and different light, and (ii) TLoL allowed students to self-reflect and to gain a sense of personal growth and recognize own individual capacities and interests.

##### 3.3.2.1 Theme 1: TLoL allowed the students to walk in a rural professional’s shoes thereby enabling them to see “rural” in a new and different light

- Students heard inspiring and motivating insights about rural medical practice.
  - *“I like to build relationships and the lifestyle benefits afforded by smaller communities are appealing. I feel inspired to provide care for people in a rural setting.”* (S41)
  - *“At lot of the messages were inspirational in motivating me to pursue this career and secondly, the appeal of rural medicine was reinforced due to the diversity of practicing it that was shared. Rural medicine is definitely appealing!”* (S38)
  - *“Learned about the value and struggle rural doctors undergo. Very inspirational and motivational. I’d love to provide more aid to those in rural areas who do not receive enough healthcare.”* (S52)
- Students found the diversity and wide scope of practice in rural practice highly appealing.
  - *“I think that the diversity of opportunity working as a doctor in rural medicine really stood out to me. There is so much you can do in rural medicine and you can tailor this career to suit your interests and needs as evidenced by the broad range of the “books” experiences.”* (S16)
  - *“The diversity of the practice is very interesting. To be able to use a* var*ied skill could be challenging but it will keep a doctor on his toes and could be satisfying. I am inspired by the stories of the books.”* (S38)
- Students learned about opportunities and flexible lifestyle that make a rural career fulfilling.
  - *“Rural medicine has a lot of different opportunities and is flexible in terms of what you want to pursue. Rural practice is not only important for a remote community, but it can also offer many different opportunities and flexibility with your career. I like the idea of being able to do a variety of skills. It can be a very rewarding career.”* (S37)
  - *“That rural medicine is a good fulfilling career. Yes, because I want to have a more meaningful as a physician.”* (S85)
  - *“That rural medicine offers many diverse opportunities for physicians to practise, and there’s a very wide scope of possibilities within rural family medicine that can suit your own particular lifestyle and goals.”* (S60)
  - *“There is a lot more to rural medicine than I initially thought. Rural medicine can be diverse and interesting and you get to practice a wide* var*iety of skills. I did not realize that a rural career offers so much flexibility. I think it can be challenging but at the same time very rewarding and fulfilling.”* (S42)
  - *“There are varied opportunities that could bring a flexible lifestyle and build relationships within the community.”* (S34)
- Students gained a better appreciation of the importance of rural physicians.
  - *“I think that rural physicians are very important in a rural community.”* (S29)
  - *“Rural medicine is under-looked …. but it is vital to keep communities healthy.”* (S15)
  - *“I have still a long way to go so I could not say for sure what I want to do right now, but I think that being a rural doctor with a diverse practice could be interesting. Not only that, there is a huge need for rural doctors. So rural doctors are important.”* (S2)
  - *“Rural physicians play an important role and this could be very rewarding in many aspects - professionally, socially, financially.”* (S20)

##### 3.3.2.2 Theme 2: TLoL allowed students to self-reflect and gain a sense of personal growth

- Students got to know their own individual capacities and interests, and potentially rural medicine.
  - *“Considering rural medicine as career. I gained new insights, which fueled my passion for medicine in rural practices.”* (S87)
  - *“Rural primary care is more personal than what we see in cities, there’s more outlets for creativity and initiative in rural medicine. Dr. Sarah’s story was interesting because I always wondered if… Would be feasible as a visible minority women.”* (S59)
  - *“As an Indigenous person, I am interested in practising rural medicine and providing health services in reserves where there typically is lack of access and funding.”* (S60)
  - *“I never thought I would consider rural medicine but now I see that it could be something I am interested in.”* (S11)
  - *“To embrace your passions and be okay with the uncertainty. Absolutely.”* (S67)
  - *“My interests align with rural medical practice.”* (S41)
  - *“Choose a field that deserves you and do not be afraid to say no to some opportunities.”* (S8)
- For some students, TLoL made them see that rural practice may not be realistic for them.
  - *“Life is not linear. This event has given me a better understanding of the scope of practice of a rural doctor. While it is fascinating, I feel it could be challenging and a bit scary, especially if you are isolated. I do not think I would be suited for this lifestyle, unless I am truly prepared for it.”* (S19)
  - *“Thanks to this event, I learned about rural medicine and what it takes to be a rural doctor. I’m not sure I’m made of the right stuff and have a practice where you are self-sufficient and self-reliant. It is such a huge responsibility. Proper training and preparation is necessary.”* (S30)
  - *“I recognized that there are many paths to medicine and practicing in rural communities can be rewarding. I think it is important that people in rural communities also receive good health care. I grew up in a big city and thus have not really considered practicing in a rural area.”* (S28)
  - *“Rural Medicine Doctors are able to develop and utilize a wide breadth of skills. I do not know much about rural medicine. I agree that rural communities need good doctors to provide medical care for them. It is admirable and inspiring that rural doctors can develop and can use a wide* var*iety of skills. I’m not sure of I’m good enough to be one.”* (S27)
  - *“Rural medicine offers a lot more than what I expected. Being from Calgary, I’m not sure how well rural medicine would integrate with my goals.”* (S53)
- Students understood better the value of connections and relationships in the community.
  - *“Advocacy is huge in rural medicine. I saw people who had similar hesitation about going rural but figured it out.”* (S74)
  - *“Rural medicine is where you can feel connected to your patients and can have a stronger and longer lasting impact on their lives. I feel that close connection to patients is important.”* (S89)
  - *“Connection to patients in rural communities. I 100% want to be in a rural community. The community aspect is so important to me.”* (S66)
  - *“Despite rural health care constantly being looked up from the deficits point of view, there are a number of assets that rural health care can provide. I would like to be a trusted member of a community who can have a positive impact on community members.”* (S96)
  - *“I want to have a great diversity of practice and love the idea of being a pillar in my community.”* (S70)
- Students gained deeper insight on the resilience and self-reliance of rural doctors in their practice.
  - *“For me resilience, passion about our careers, and being open minded. I think words are powerful, and hearing some of the stories and even reassurances has changed my lens on rural medicine.”* (S71)
  - *“Hearing about all these resilient stories was truly eye-opening. Every single one of these successful “books” had a fluctuating, up and down bumpy road, and I feel inspired to never give up if there’s passion.”* (S41)
  - *“Do not forget to be resilient and even if you want to start over and reinvent yourself, that is okay.”* (S34)

## 4 Discussion

The goal of this study was to determine whether TLoL is an effective learning strategy in medical education for changing the perceptions of learners about rural life and rural practice. After completing three TLoL events, sufficient quantitative and qualitative data about the students seem to support the research inquiry. The data showed that only 20% of the students are from a rural background, which is consistent with recent research (24) showing that Canadian medical students are more likely to have urban upbringing. This potentially reflects why the data also indicated that the students have low familiarity with rural medicine, with only 18% expressing familiarity with rural practice. Despite this, it is encouraging that almost all indicated a keen willingness to learn more about rural practice. The pre-TLoL qualitative data demonstrated that students were not only curious about rural medicine but were also eager to make connections to the human books, to engage with them in open dialogue and to hear about their personal experiences, challenges and struggles. Additionally, students looked to seek professional advice and guidance from the books, especially about what it takes to be a rural healthcare professional.

The statistical analysis of quantitative data revealed significant improvements in attitudes and perceptions scores from pre- to post-TLoL experience. This suggests that TLoL has a meaningful impact on changing students’ attitudes and perceptions about rural medicine. Positive changes were observed in terms of students’ attitudes about rural medicine as a potential future career option, appreciation of rural life and work, and recognition of the need for and importance of rural physicians. In particular, TLoL showed positive effect on rural practice consideration, with a marked improvement from 44% to 72% of students indicating they could potentially see themselves engaged in a similar rural medical practice.

This is reinforced by the qualitative findings and summarized by two overarching themes. The first main theme was that TLoL allowed the students to walk in a rural professional’s shoes thereby enabling students to appreciate “rural” under a fresh and newfound light. Inspired by the books’ motivating stories and experiences, the students gained valuable insights about rural medical practice and found the diversity and wide scope of practice in rural practice highly appealing. Further, students learned that the diversity and flexible lifestyle are what makes a rural career fulfilling and rewarding. Seen under this new light, they were able to discern the important and vital role of the rural physician. The second main theme was that TLoL allowed students to self-reflect and gain a sense of personal growth. Students got to know their own individual capacities and interests, and potentially rural medicine; they gained deeper insight on the resilience and self-reliance of rural doctors in their practice and the value of building relationships with patients and within the community. However, this self-reflection can also lead students to comprehend that rural practice may not be realistic for them. These themes describe the elements that influence the mechanism of the change in perception and attitude toward rural practice.

The change in attitude toward rural practice can be likened to a change in behavioral intention (e.g., the willingness to communicate and cultivate a relationship with, and cater support for an underserved community), which is believed as one of the most difficult to effect change among attitudinal factors (44). The finding suggests that students made gains in self-perceived knowledge about rural medicine and in attitudes toward rural practice through the self-reflection and personal connections offered by TLoL. Thus, the results show the potential power of narratives to change human perceptions. The findings are consistent with other research demonstrating the impact of experiential learning offered by the living library approach in various healthcare professions (32–35). As such, educators and learning institutions are increasingly using the living library concept as an alternative learning modality.

The living library is not only a novel but also a flexible approach in medical education (3). The logistics of TLoL differed between the online and in-person formats. The online TLoL allowed for the interactions between human books and students to be recorded, for the books’ stories to be viewed and shared with other people long after the event was done. On the other hand, the in-person TLoL allowed for direct eye-contact and face-to-face interaction, which facilitated deeper and more personal connection between human books and students, giving the in-person TLoL slightly more edge over virtual format. During in-person TLoL, both students and human books relished the chance to meet new people and network, enjoy the refreshments and just mingle. Further, students took the chance to ask the human books for career advice and guidance and to seek answers to their own questions. While both formats have advantages and challenges, these demonstrated the versatility of TLoL.

The authors are from a medical school that offers both culturally immersive rural block rotations and the longer longitudinal clinical clerkship experiences to medical students. TLoL presents students with a glimpse of what to expect in rural living and can encourage a healthy interest and foster positive attitudes toward rural experiences and to consider these rural learning opportunities. One student said that during their TLoL experience they did not feel like they were being lectured at but rather merely having an open conversation, so they felt more engaged and learned more. Another student proposed that something like this (TLoL) be integrated into the medical school curriculum. This suggests that students found TLoL a safe space for engagement and learning. The culturally safe space and opportunity for self-reflection are the essential attributes that may make living libraries effective. When carefully planned and curated with relevant books, TLoL can be a suitable learning modality in medical education, to challenge attitudes and perceptions, in areas where there may be knowledge gap, such as rural medical practice.

### 4.1 Limitations

The results of this study represent data from students from one medical school in Canada. While the authors advertised our TLoL offerings well in advance, participation may have been conditional on students’ availability at the time of TLoL, i.e., only available students were able to attend. Likewise, students who may have an inherent interest in rural medicine may have been enticed to take part. Different sets of human books participated at each of the three TLoL offerings held. Each human book had their own individual story and storytelling style. Student “readers” were assigned randomly to three human books each, and each student was able to engage with a different set of human books. There were no data collected that will allow comparison of experiences of TLoL conducted virtually and as an in-person event. The results were not adjusted by event offering or by specific human books that students engaged with. No demographic groupings were considered or compared in the analysis. Therefore caution is needed in interpreting the results.

## 5 Conclusion

The study findings confirm that most students going into medicine are urban-origin and have little familiarity with rural life and rural practice. While the majority of students initially would not consider rural medicine, the results show that the use of narratives and storytelling can challenge held beliefs around rural practice and life, consistent with the original aims of the Human Library©. Through TLoL, students may be encouraged to consider things that traditional medical teaching may not offer. It is difficult to change perceptions and attitudes, but narratives can have the power to create change. The students gained a better understanding of rural practice and rural life, the situation of the books, and different beliefs in general, and received answers to their own questions and doubts. TLoL is akin to an experiential learning space and can be an effective learning modality in medical education to provide information and inspiration to learners to consider a career in rural medicine. This approach can be used in conjunction with other rural educational learning opportunities such as rural block rotations and longitudinal clinical clerkship immersions to enhance learning. The results suggest that narratives may help to facilitate and foster more favorable attitudes toward rural medical practice among our students. Implications for future research may include investigations into the actual behaviors following practice intention of students and impact of TLoL for the longer term. However, longitudinal studies can be much more challenging to conduct.

## Data Availability

The datasets presented in this study can be found in online repositories. The datasets can be found in the Open Science Framework repository [DOI: 10.17605/OSF.IO/5FCMY].
